# An ecological conception of professional coaching for newly recruited midwives: The case of maternity units in northern Morocco

**DOI:** 10.18332/ejm/169756

**Published:** 2023-09-12

**Authors:** Najat Boucetta, Mustafa El Alaoui

**Affiliations:** 1 Higher Institute of Nursing Professions and Health Techniques, Tetouan, Morocco; 2 Research Team in Pedagogical Engineering and Science Didactics, Higher Normal School, Université Abdelmalek Essaadi, Tetouan, Morocco

**Keywords:** needs, coaching, expectations, professional, conditions, newly recruited midwives

**Dear Editor,**

Maternal and newborn mortality is a pressing global public health issue, representing one of the most significant health disparities worldwide and serving as an indicator of health system shortcomings. It poses both a human rights concern and a challenge to achieving fair socio-economic development^1^. Extensive endeavors have been undertaken to address this problem, with the Sustainable Development Goals aiming to decrease maternal deaths^2^. In Morocco, maternal and child health has long been a key national priority. The Moroccan authorities have implemented strategies to enhance healthcare accessibility and improve the well-being of mothers and children in particular^3^. Efforts to reduce maternal and neonatal mortality in Morocco focus on strengthening the capacities and roles of midwives. Nonetheless, the midwifery profession is demanding, requiring responsibility and proficiency to ensure the provision of safe and high-quality care. Evidence supports the notion that obtaining appropriate academic midwifery qualifications can have a positive impact on maternal and neonatal outcomes^4^. Licensed and regulated midwives who graduate from high-quality midwifery education programs play a crucial role in countries burdened with high maternal mortality rates^5^. The objective of this study was to investigate the working conditions, needs, and expectations of professional coaching for newly recruited midwives in northern Morocco.

Our research is based on a descriptive and exploratory study conducted in maternity facilities in northern Morocco from 1 January to 31 May 2022. The study sample comprised 69 newly recruited midwives with less than 2 years of professional experience who voluntarily participated in the study. Data were collected using a questionnaire adapted from the national guide titled ‘Midwifery Supervision in Rural Health Centres’^6^ jointly developed by the Moroccan Ministry of Health and the United Nations Population Fund. The questionnaire encompassed three sections: the first section gathered general information, the second section focused on working conditions, and the third section explored the participant needs regarding professional coaching. The questionnaire was shared with the participants during a virtual meeting. Data analysis was performed using Excel 2016.

The surveyed novice midwives were aged 20–26 years, with an average age of 23 years. In terms of marital status, 45 were single and 24 were married. The analysis of their working conditions revealed a heavy workload reported by 62% of the respondents, which is consistent with findings from other studies due to the demanding nature of maternity services, known to be among the most intense and demanding fields of healthcare^7^.

**Figure 1:**
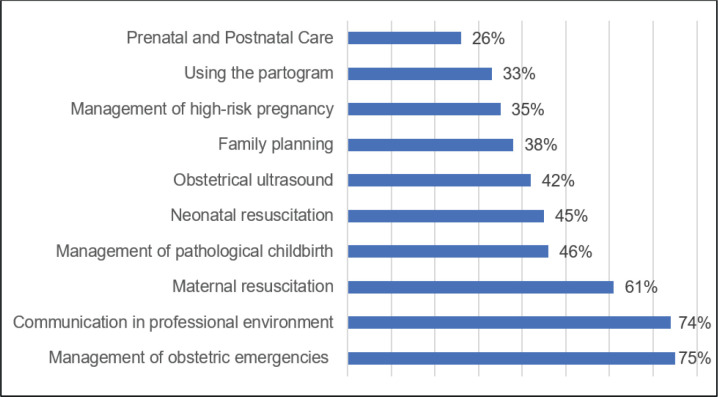
Distribution of surveyed newly recruited midwives based on the required training subjects (N=69)

Regarding workplace stress, all the surveyed newly recruited midwives experience varying degrees of stress. Jain et al.^8^ link this phenomenon to factors such as lack of experience, heavy workload, high responsibilities, dealing with new situations, and perceived deficiencies in organizational skills, which contribute significantly to this issue. A notable 84% of the surveyed midwives expressed a need for training. Specifically, 75% required training in managing obstetric emergencies, 74% sought to enhance their communication skills in a professional environment, 61% required training in maternal resuscitation, 46% needed guidance in managing pathological childbirth, 45% sought knowledge in neonatal resuscitation, 42% desired training in obstetrical ultrasound, 38% required training in family planning, 35% needed expertise in managing high-risk pregnancies, 33% desired training in utilizing the partogram, and 26% sought training in prenatal and postnatal care (Figure 1).

Furthermore, the data indicate that 83% of the midwives expected psychological support, 78% anticipated assistance in problem-solving, 59% looked forward to guidance in decision-making, 57% sought assistance in developing technical skills, and 54% expected support in assuming responsibility. Work-related stress is believed to stem from a lack of motivational mechanisms, ill-defined work tasks, conflicts and tensions, an absence of teamwork, and inadequate feedback from superiors. The workload, coupled with the absence of professional coaching, hinders newly recruited midwives from working calmly and fully utilizing their knowledge and skills. The findings of this study underscore the necessity of supporting and assisting newly recruited midwives. Identifying their working conditions, addressing their needs through a system of nearby support, and offering updated training based on active pedagogical approaches are essential. These measures aim to enhance their commitment and motivation, facilitating a smooth transition from the learning phase to their professional careers.
